# Impacts of hydrogeochemical processes and land use practices on groundwater quality of Shwan sub-Basin, Kirkuk, northern Iraq

**DOI:** 10.1016/j.heliyon.2023.e13995

**Published:** 2023-02-26

**Authors:** Hind Fadhil Al-Gburi, Balsam Salim Al-Tawash, Omer Sabah Al-Tamimi, Christoph Schüth

**Affiliations:** a Geology Department, College of Science, University of Baghdad, Baghdad, Iraq; b Department of Applied Geology, College of Science, University of Kirkuk, Kirkuk, Iraq; c Institute of Applied Geosciences, Technische Universität Darmstadt, Darmstadt, Germany

**Keywords:** Hydrogeochemistry, Shwan sub-Basin, Groundwater, Saturated index, Water quality index

## Abstract

Shwan sub-Basin is one of the substantial groundwater sources in northern Iraq. Along with an increase in population, agricultural and industrial activities synced with the change in climate conditions, all could have a negative impact on the hydrochemistry of groundwater. Therefore, it becomes crucial to investigate the different processes that could affect hydrochemistry and water quality. Thirty-two groundwater samples were collected from wells distributed in the study area, and one surface water sample from Lesser Zab River, all water samples were gathered during two seasons. Hydrogeochemical model was performed on physiochemical analysis results by using PHREEQC software to understand the geochemical reactions occurring in groundwater. The results of the Saturated Index showed supersaturated values for calcite, aragonite and dolomite in groundwater samples during the first season in a percent of 84%. While the second season samples were supersaturated in percent of 40.6%, 37.5% and 46.8% for aragonite, calcite and dolomite minerals respectively. The Saturated Index shows supersaturated values of quartz mineral in most groundwater samples, which are sourced from the abundance of silicate minerals that are primarily included within the ambient rock materials of the tertiary and quaternary clastic aquifer system in the study region. The saturated index showed undersaturated values with most minerals of feldspar, halide and sulfate. However, these minerals were in a dissolution state, releasing significant amounts of Ca^2+^, Na^+^, Mg^2+^, HCO_3_^−^, Cl^⁻^ and SO_4_^2−^ ions into the solution. Most of the groundwater samples were classified as earth-alkaline water with an increased portion of alkali with prevailing bicarbonate for two seasons, except the groundwater sample W2 was classified as earth-alkaline water with an increased portion of alkali with prevailing SO_4_^2⁻^ and Cl^⁻^. The water quality for human drinking was evaluated using the water quality index (WQI). The values of WQI were from 51.9 to 99.2 and from 53.9 to 88.5 for the first and the second seasons respectively. WQI revealed that most of the samples were classified as poor to very poor water quality, except the Lesser Zab River sample for the second season was good water quality and the sample W2 for the first season was unsuitable for drinking purposes.

## 1 Introduction

Groundwater quality is determined in natural settings by the total weathering of rock by the interaction between rocks and groundwater in hydrogeologic aquifers [1]. For several elements, the initial concentrations and ratios are very similar to the parent geology. The fundamental groundwater characteristic is thus provided by aquifer lithology and mineral assemblage, and geochemical reactions in groundwater (dissolution-precipitation, ion-exchange, or redox reactions) [1]. Hydrogeochemical studies are considered one of the important tools to identify and control the interaction processes of groundwater with aquifer minerals [2]. Geochemical models based on equilibrium thermodynamics have been widely used to interpret field data and to provide theoretical descriptions for the origin and evolution of natural systems [3]. The PHREEQC geochemical model was used to predict groundwater geochemical processes such as dissolution, precipitation reactions, and mixing of diverse water supplies [4]. PHREEQC was utilized in several studies to analyze the transport and change of chemical inorganic species caused by precipitation or dissolution processes [[5], [6], [7]]. The geochemical models can be effective tools for exploring groundwater chemistry and the possible chemical behavior of mineral species under specific hypothetical conditions [8]. The majority of groundwater is originating directly from rainfall infiltrating the land surface. Thereby, Land Use Land Cover (LULC) has a significant impact on both groundwater quality and recharge rates. Regardless of climatic conditions, different land-use practices leave distinct signatures on the quality of groundwater recharge and, in some cases, result in diffuse groundwater pollution. Similarly, land-use practices have an impact on groundwater recharge rates, particularly in more arid conditions [9]. Groundwater pollution may clearly appear when looking at specific substances introduced as pollutants (tracers) and non-existing (to some extent) from baseline, especially those of fully artificial origin [10]. However, groundwater quality assessment considers one of the substantial measures in regions with high vulnerability to pollution by land use practices [11]. Several studies adopted the water quality index (WQI) to evaluate the water quality for human drinking [[11], [12], [13], [14], [15], [16]]. The Water Quality Index (WQI) is a statistical method that converts enormous quantities of description data into a single value that represents the water quality [15,17]. Surface water shortages are now the most critical situation in arid and semi-arid climate environments around the world [18]. As a result, groundwater is regarded as a significant viable alternative resource in these environments [19,20]. The Middle East, an arid to semi-arid region, is the world's most water-scarce zone, with the majority of its countries falling under the United Nations' international water poverty index [21].

Iraq is one of the Middle Eastern countries experiencing severe water shortages [22]. Iraq is highly reliant on the Tigris and Euphrates rivers, however, due to drought and upstream damming, these rivers' discharges have declined in the last decades, consequently a growing need for alternate water resources [23]. The dependence on groundwater is common in most regions of northern Iraq as an effective water resource [6,[24], [25], [26]]. Shwan sub-Basin is located in northern Iraq, it relies on groundwater as a major source of water supply for drinking, domestic, irrigation, and industrial purposes [26,27]. Furthermore, since it might be a promising water resource in the Kirkuk governorate, the main geological processes that control the hydrochemistry of groundwater in the study area should be studied and identified thoroughly cause the groundwater chemistry in the study region could be affected by leaching, dissolution, precipitation, and ion exchange processes of aquifer minerals. In addition, land use practices such as agriculture and industrial activities that were recently increased in the Shwan sub-Basin can contribute to the change of hydrochemical properties of groundwater. Therefore, the aims of this study are (1) to identify hydrogeochemical processes of the groundwater aquifer using the PHREEQC model (2) to assess the effect of the different land use practices on the hydrochemistry of the groundwater; (3) to study the feasibility of groundwater usage for human drinking based on WQI.

### 1.1 Study area

**Fig. 1:**
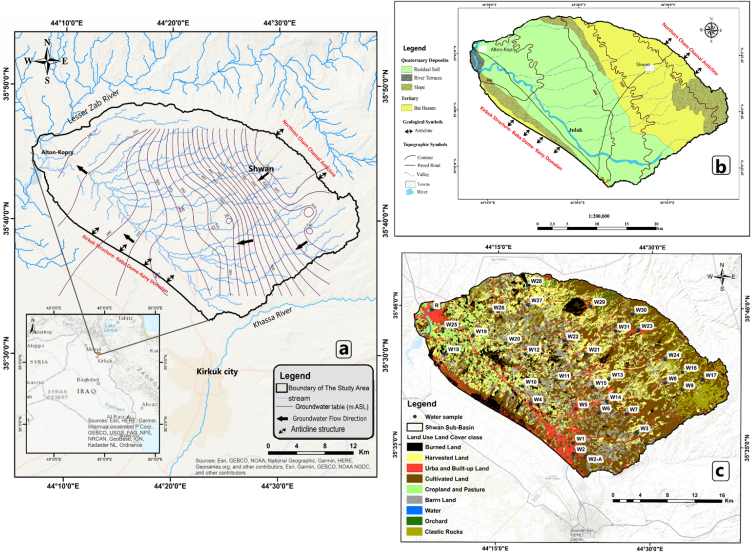
Location of the study area (a), Geological formations at the study area (b) [30], LULC map and water sample sites (c).

The study area is located north of Iraq, northeast of Kirkuk Governorate and occupies an area of 829 km^2^ (Fig. 1 a). The topography of the study area varies from low elevated to semi-flat terrain with elevation ranges between 200 and 850 m above sea level (ASL). The agricultural activities represent the mainland practices (Fig. 1c). In addition, other land uses such as industrial activities that located in the southern and western parts of the study area. The exposed geological Formations have an age range from Miocene to Holocene [28] (Fig. 1b). Bai-Hassan Formation was exposed to the basin's northeast along the northern Cham Chamal Anticline. However, the Quaternary deposits (Pleistocene and Holocene) covered the center of the basin [29]. Confined and unconfined aquifers of Quaternary deposits and Bai-Hassan are prolific hydrogeological units in the studied area (Fig. 1b), consisting of clastic sediments of alternating sandstone and gravel beds with clay and conglomerate beds [26,27]. All wells in the basin penetrate the Bai-Hassan formation to some extent. The groundwater flow direction in the study area is from east to southeast to northwest (Fig. 1 a) [26].

### 1.2 Climate of the study area

The study area has a Mediterranean climate, distinguished by cold winters with little rain and arid hot summers [31]. For the last four decades, 1981–2021, meteorological data [32] indicate that the total annual rainfall for the last four decades was 325.43 mm, and the monthly mean temperature is 23.05 °C. The mean relative humidity is 37.17%, the mean wind speed is 2.49 m/s, and the mean annual evaporation is 429.55 mm. On the other hand, the results of observed meteorological data for the sampling year (2020–2021) show a very little amount of precipitation with an annual rainfall of 100.62 mm.

## 2 Materials and methods

### 2.1 Materials

#### 2.1.1 Sampling

Thirty-two groundwater samples and one surface water sample from Lesser Zab River (LZR) were collected from the study area for two seasons (Fig. 1 c). The first season was in October 2020, and the second season was in April 2021. From each sampling location, two water samples were collected in polyethylene bottles (500 mL). One of the two samples was filtered and acidified to pH ≈ 2 with concentrated nitric acid (HNO_3_) to preserve samples for cation and heavy metal analyses. Besides, two more samples were collected in glass amber bottles one for biological oxygen demand (BOD) analyses (500 mL) and one for dissolved organic carbon (TOC), then all samples were stored in a cooler before being transported to the laboratory.

#### 2.1.2 Sample analyses

Physiochemical parameters of water temperature (WT), turbidity, pH, electrical conductivity (EC), total dissolved solids (TDS), salinity, dissolved oxygen (DO) and oxidize redox potential (ORP) were measured in the field (during the first and second sampling seasons) for all water samples that have been sampled from Shwan sub-Basin by using a portable multimeter. Whereas the other chemical characteristics of the water samples were analyzed in the lab according to the methods of the American Public Health Association (APHA) [33]. Bicarbonate (HCO_3_^−^) was determined via titration method using an indicator titrated with HCl.

Biological oxygen demand (BOD) was determined according to the five-day BOD test [34], and the colorimetric method [35] was used to determine chemical oxygen demand (COD), The dissolved organic carbon (DOC) determined with Liqui TOC (elementary analysis system GmbH). Silicon (Si) in water samples was determined by using a spectrophotometer [36].

Water samples were sent to the Institute for Applied Geosciences, Technische Universität Darmstadt, Germany to be analyzed by inductively coupled plasma mass spectrometry (ICP-MS)**,** samples were analyzed with an Analytik Jena plasma quant MS Elite. The analytical procedure for heavy metals was conducted by preparation of 2% HNO_3_ solution (dilution solution): 55 mL HNO_3_ (69%, supra-quality) in 4000 mL Milli-Q water, while preparation of tuning solution [1 μg/L; 1 ppb]: 10 μL. Tuning solution in 100 mL dilution solution. Preparation of internal standard solution [10 μg/L; 10 ppb]: 50 μL of AJ internal standard in 500 mL dilution solution (or addition of 7.5 mL HNO_3_ (69%, Supra-Quality) and fill up with Milli-Q water).

The analytical precision for cation and anion analyses for the two seasons was evaluated by verifying ion balance errors with PHREEQC software version 3.1.48,929 [37]. The errors were less than 5% and varied from −1.9 to 3.2%.

### 2.2 Methods

#### 2.2.1 Geochemical speciation models of groundwater chemistry

The geochemical speciation model was applied to the results of groundwater analyses to identify different variables as saturation indices of selected minerals phases and the partial pressure of CO_2_ gas using PHREEQC software version 3.1.48,929. Information of mineral saturation states is a benefit for interpreting minerals that control on the concentrations of ions and for understanding probable chemical reactions for mass-balance modeling. The dimensionless SI is defined as the logarithm of the ratio of ion activity product (IAP) to the mineral equilibrium constant at a given temperature. The ion activity product (IAP) has the same form as the equilibrium constant (Ksp) but involves the actual activities. The ratio between IAP and Ksp enters the definition of the saturation index. The SI is calculated according to equation (1) [30].

(eq. 1) $$SI=\mathrm{Log}\;\frac{IAP}{Ksp}$$

Moreover, rock–water interaction [38] and classification of water type were utilized to understand and differentiate the influences of rock-water interaction, evaporation and precipitation on water chemistry [39].

#### 2.2.2 Water suitability for human drinking

Comparison of different water parameters with quality guidelines of Iraqi standard (IQS) and WHO [[40], [41], [42]], were used as the basis for the water quality evaluation of the present study water samples for drinking use. The suitability of water for agriculture is evaluated depending on the criteria or standards of acceptable quality for that use.

Water Quality Index (WQI) is a numerical expression that provides the composite influence of numerous parameters on the overall water quality, it's the perfect indicator for water quality [[11], [12], [13]]. Furthermore, WQI is used to assess the suitability of water quality for human drinking purposes [[14], [15], [16]]. WQI is calculated by applying the following equations (eq. (2), eq. (3), eq. (4), eq. (5)) [11]:

(eq. 2) $$Wi=\frac{K}{Si}$$

(eq. 3) $$K=\frac{1}{\sum_{i}^{n}=\frac{1}{1Si}}$$

(eq. 4) $$Qi=\left[\frac{V\left(-\right)Vi}{Si-Vi}\right]\;*100$$

(eq. 5) $$WQI=\frac{\sum_{i}^{n}=1WiQi}{\sum_{i}^{n}=1Wi}$$

Where: *Wi* is unit weightage of *i*th parameter (Table 1), *K* is the proportionality constant, *Si* is the standard value of *i*th parameter (Table 1), *Qi* is the sub-index of the *i*th parameter, *V* and *Vi* the observed value and ideal value (the ideal value is equal to zero, except *Vi* for pH is 7) of *i*th parameter. The computed WQI values were classified into five classes: <25 Excellent water, 26–50 Good water, 51–75 Poor water, 76–100 Very poor water and >100 Unsuitable for drinking [11].

**Table 1:** Relative weight of chemical parameters.

Parameter	Unit	WHO, Standard	1/sn	∑1/sn	K = 1/∑1/sn	Wi = K/Si
pH		8.5	0.12	5.59	0.18	0.021
**Turbidity**	(NTU)	5	0.2	5.59	0.18	0.0358
**Alkalinity**	mg/L	200	0.005	5.59	0.18	0.0009
**Hardness**	mg/L	500	0.002	5.59	0.18	0.0004
**BOD**	mg/L	5	0.2	5.59	0.18	0.0358
**COD**	mg/L	10	0.1	5.59	0.18	0.0179
**Ca**^**2+**^	mg/L	100	0.01	5.59	0.18	0.0018
**Mg**^**2+**^	mg/L	125	0.008	5.59	0.18	0.0014
**Na**^**+**^	mg/L	200	0.005	5.59	0.18	0.0009
**K**^**+**^	mg/L	12	0.08	5.59	0.18	0.0149
**HCO**_**3**_^**−**^	mg/L	200	0.005	5.59	0.18	0.0009
**SO**_**4**_^**2-**^	mg/L	250	0.004	5.59	0.18	0.0007
**Cl**^**−**^	mg/L	250	0.004	5.59	0.18	0.0007
**NO**_**3**_	mg/L	50	0.02	5.59	0.18	0.003
**NO**_**2**_	μg/L	3	0.33	5.59	0.18	0.059
**PO**_**4**_	μg/L	0.4	2.5	5.59	0.18	0.44
**Al**	μg/L	100	0.01	5.59	0.18	0.0018
**As**	μg/L	10	0.1	5.59	0.18	0.0179
**Ti**	μg/L	100	0.01	5.59	0.18	0.0018
**V**	μg/L	6	0.16	5.59	0.18	0.0298
**Cr**	μg/L	50	0.02	5.59	0.18	0.0036
**Mn**	μg/L	80	0.012	5.59	0.18	0.0022
**Fe**	μg/L	300	0.003	5.59	0.18	0.0006
**Co**	μg/L	50	0.02	5.59	0.18	0.0036
**Ni**	μg/L	70	0.01	5.59	0.18	0.0026
**Cu**	μg/L	2000	0.0005	5.59	0.18	0.0001
**Zn**	μg/L	3000	0.00033	5.59	0.18	0.0001
**Se**	μg/L	40	0.025	5.59	0.18	0.0045
**Mo**	μg/L	70	0.014	5.59	0.18	0.0026
**Cd**	μg/L	3	0.33	5.59	0.18	0.0596
**Sb**	μg/L	20	0.05	5.59	0.18	0.0089
**Ba**	μg/L	700	0.0014	5.59	0.18	0.0003
**Pb**	μg/L	10	0.1	5.59	0.18	0.0179
**Th**	μg/L	1	1	5.59	0.18	0.178
**U**	μg/L	30	0.033	5.59	0.18	0.006
**Total**			5.59	5.59	0.18	1

## 3 Results and discussion

### 3.1 Hydrochemical measurements

**Fig. 2:**
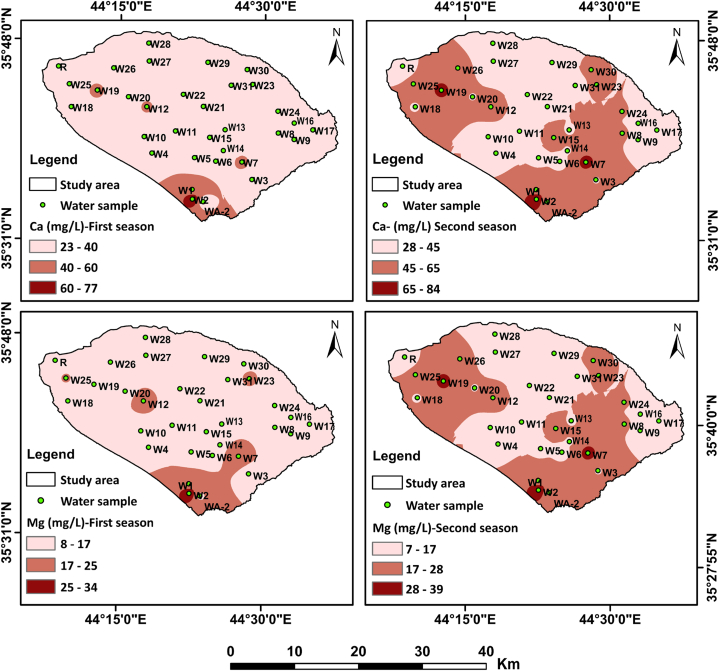
Spatio-temporal maps of cations in water samples.

**Fig. 3:**
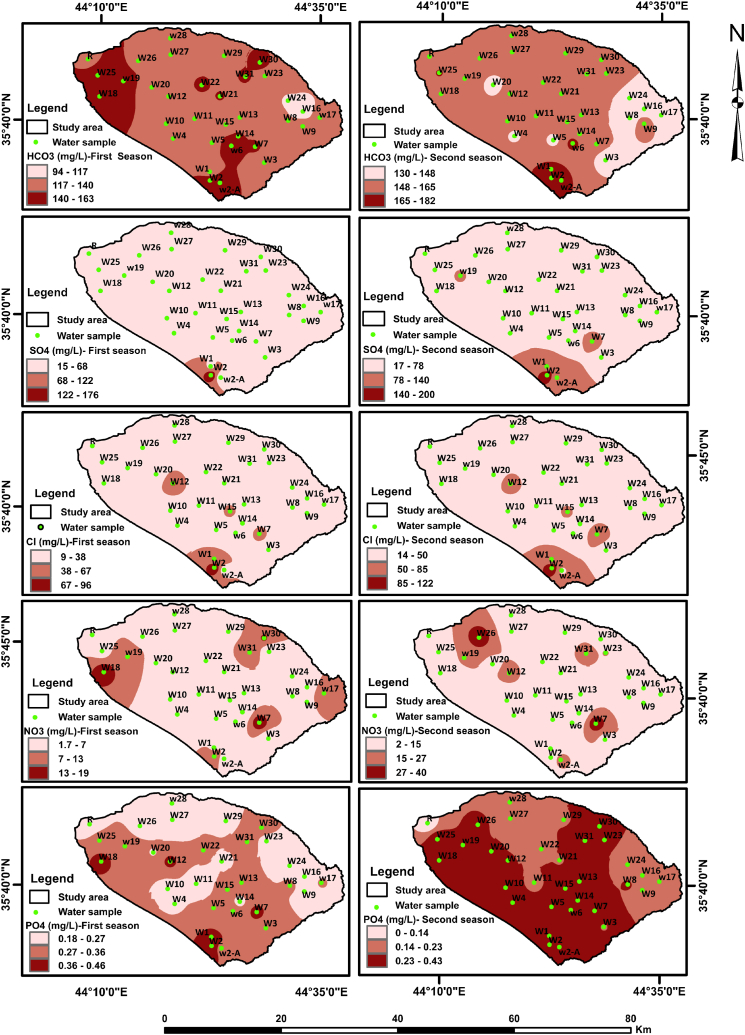
Spatio-temporal maps of anions in water samples.

Fig. 2, Fig. 3 showed spatio-temporal maps that show the intrinsic major and minor variables of the groundwater and LZR samples. The varied and complex response of different chemical variables in the groundwater samples shows interesting patterns of noticeable variations in groundwater hydrochemistry. Which showed a wide range of seasonal and spatial variability in the solute concentrations of groundwater samples. This is explained by changes in hydrological and lithological characteristics.

In addition to various natural processes, this study reveals the significant impact of human activities on the hydrochemical characteristics of groundwater. The noticeable variations in the spatial distribution pattern of most elements were in the south and northwest parts of the sub-Basin (Figs. 2 and 3) and samples correspond to an abrupt change in the lithology as well as anthropogenic activities. The average abundance of the major ions in groundwater follows the decreasing order: HCO_3_^−^ > SO_4_^2−^> Ca^2+^> Cl^−^> Na^+^> Mg^2+^> K^+^ during two sampling seasons (Table 2).

**Table 2:** Summary statistics of physiochemical and biological parameters in water samples from Shwan sub-Basin for two sampling seasons.

Parameter	First season						Second season					IQS (2009)	WHO,2008 and 2017
	Unit	Min	Max	Mean	SD	LZR sample	Min	Max	Mean	SD	LZR sample		
A. T.^a^	°C	16	18.3	17.1	0.69	18	22	34.1	27.9	3.77	31		–
W.T.^b^	°C	16.3	25.1	21.3	2.06	21.5	21.3	29.8	24.9	2.02	24		–
pH		7.1	8.4	7.6	0.3	8.2	7	7.8	7.4	0.2	8	6.5–8.5	6.5–8.5
TDS	mg/L	260	518	308	67.6	202	223	772	340	107	227	1000	1000
EC	μs/cm	402	826	465	102	413	341	1170	519	164	332	–	1530
Salinity	mg/L	199	508	246	71.3	272	166	583	260	81.8	173	–	–
Turbidity	(NTU)	4	10	7.18	1.67	12	3	10.7	7.55	1.88	11.2	–	5
DO	mg/L	3.5	13.2	9	2.44	18	2.83	5.06	3.96	0.51	4.76	–	–
ORP	mv	−78	−7.6	−36.2	16.6	−66.2	−42	38	−20.5	15.2	−17	–	–
DOC	mg/L	0.89	1.86	1.27	0.23	1.81	0.78	1.28	1.02	0.14	0.82	–	4.58^c^
BOD	mg/L	1.3	7	3.90	1.28	2.9	3	7	4.91	0.87	8.5	<5	5
COD	mg/L	90	175	112	21.5	100	90	160	120	18.7	175	<5	10
Alkalinity	mg/L	60	160	119	25.8	135	40	200	138	40.6	110	–	200
Hardness	mg/L	60	245	143	51.6	60	40	260	128	54.3	40	500	500
Ca^2+^	mg/L	23	77	35.3	9.45	32	28	84	43	12.1	37	150	100
Mg^2+^	mg/L	8.21	34	14.8	4.86	12.7	6.7	39	17.9	6.52	14.4	100	125
Na^+^	mg/L	7	56	19.1	8.86	27	9	69	24.5	11.7	29	200	200
K^+^	mg/L	0.22	2.8	0.80	0.55	0.49	0.47	4	1.38	0.78	0.38	–	12
HCO_3_^⁻^	mg/L	94	163	135	14.2	130	131	182	154.5	11.7	150	–	200
SO_4_^2⁻^	mg/L	15	176	41.3	28.2	48	17	200	54.5	34.4	54	400	250
Cl^⁻^	mg/L	9	96	24.1	18.2	19	14	122	33.7	24.3	23	350	250
Si	mg/L	9.75	15.4	12.9	1.32	11.3	10	15.7	13	1.22	14.2	–	–
NO_3_	mg/L	1.8	19	6.7	4.61	1.7	2	40	10.8	9.31	4.2	50	50
NO_2_	mg/L	ND	0.2	0.04	0.05	ND	ND	0.7	0.16	0.19	ND	3	3
CO_3_	mg/L	ND	0.91	0.48	0.32	ND	0.18	0.81	0.45	0.17	ND	–	–
PO_4_	mg/L	0.18	0.46	0.29	0.06	0.19	0.16	0.43	0.30	0.07	ND	–	0.4
Well depth	Meter	80	180	–	–	–	–	–	–	–	–	–	–
Water table	Meter	13	80	–	–	–	–	–	–	–	–	–	–

a Air temperature.

b Water temperature.

c DOC mean value in European groundwater [1].

Calcium (Ca^2+^) ion has the highest value among the other cations in all groundwater samples except W27 and W28 for the first and second seasons. The highest concentrations of HCO_3_^⁻^ in the sub-Basin samples are found in samples W18, W2 and W6 for the first, while W2 and W6 detected the highest concentrations for the second seasons (Fig. 2, Fig. 3).

The Maximum value of the most chemical ions was detected in groundwater sample W2 for two sampling seasons (Fig. 2, Fig. 3). This is due to the effect of the petroleum refinery which is located close to this well location. The highest Cl^−^ concentrations were found in W2, W12, W7 and W15 for the first season, whereas the highest Cl^−^ concentrations were found in W2, W7, W12, and W15 for the second season (Fig. 2). The effects of land use are the main reason behind the highest Cl^−^ concentrations in these wells. For the W2 well the effect of the waste from the petroleum refinery. Wells W7 and W15 have been affected by the infiltration of pollutants from local unsealed septic tanks that were close to these wells. The effect of fish farming was behind the raising in Cl^−^ concentrations in the W12 well.

In general, TDS, EC, major and minor ions, as well as heavy metals, exhibit higher concentrations in groundwater samples and LZR samples during the second season than concentrations in the first season (Table 2, Table 3, and Fig. 2, Fig. 3, Fig. 5). Such an increase pattern may due to the dry weather conditions from high air temperatures, evaporation and little precipitation [43], which led to a low in the amount of recharge to groundwater during the study period. Thus, more solute concentrations are released to groundwater during the resident time and along its flow path as a result of dissolution processes within the aquifer system see Table 2, Table 3.

**Table 3:** Summary statistics of heavy metals in the groundwater samples and LZR sample for two sampling seasons.

Element	Unit	First season				Second season					
		Min	Max	Mean	LZR	Min	Max	Mean	LZR	IQS (2009)	WHO, 2017
**Al**	**μg/L**	0.13	15	5.98	458	4.93	100	15.4	85.4	100	100
**As**	**μg/L**	0.38	1.12	0.76	1.88	0.49	1.11	0.76	1.29	10	10
**Ti**	**μg/L**	0.01	0.6	0.15	0.35	0.03	1.66	0.38	0.84	–	100
**V**	**μg/L**	3.47	13	9.46	4.00	3.44	17.7	10.5	1.81	–	6
**Cr**	**μg/L**	0.32	15.9	4.68	3.07	1.62	16	5.47	6.38	50	50
**Mn**	**μg/L**	0.12	9.06	1.45	339	0.15	8.49	1.50	22.5	100	80
**Fe**	**μg/L**	3.19	449	56.9	315	2.29	279	46.3	110	300	3000
**Co**	**μg/L**	0.02	0.15	0.04	1.60	0.01	1.79	0.11	0.25	–	50
**Ni**	**μg/L**	0.09	5.53	1.46	11.1	0.15	5.45	1.72	6.47	20	70
**Cu**	**μg/L**	0.01	4.21	0.51	1.50	0.02	2.91	0.71	1.35	1000	2000
**Zn**	**μg/L**	0.41	571	70.6	5.22	2.69	675	51.9	11.9	300	3000
**Se**	**μg/L**	0.21	1.23	0.58	0.17	0.25	1.17	0.60	0.45	10	40
**Rb**	**μg/L**	0.13	0.54	0.28	0.64	0.15	0.58	0.33	0.59	–	–
**Mo**	**μg/L**	0.72	7.26	2.09	0.06	1.25	8.08	2.60	1.82	–	70
**Cd**	**μg/L**	0.01	0.02	0.01	0.06	0.01	0.02	0.01	0.02	3	3
**Sn**	**μg/L**	0.01	0.04	0.01	<0.01	0.01	0.07	0.02	0.03	–	1–2
**Sb**	**μg/L**	0.12	1.13	0.81	1.13	0.01	1.45	0.39	1.47	–	20
**Ba**	**μg/L**	25.3	155	72.8	69	31	168	74.7	39.8	–	700
**Pb**	**μg/L**	0.01	0.82	0.29	1.59	0.01	1.99	0.53	0.55	10	10
**Th**	**μg/L**	0.001	0.01	0.01	<0.001	<0.001	0.01	–	<0.001	–	0.01–1
**U**	**μg/L**	0.42	4.49	1.57	0.50	0.46	4.45	1.63	0.46	–	30

#### 3.1.1 Biological characteristics

Dissolved Oxygen (DO), BOD, and DOC were tested in water samples, and the ranges and means of measurements in groundwater and LZR samples are presented in Table 2. The rate of dissolved oxygen elimination is highly influenced by the aquifer's geochemistry and the availability of electron donors (most notably dissolved organic matter - total organic carbon [TOC] or Fe^2+^ present in pyrite or, to a lesser extent, silicates). Fast-moving water, low temperature, and low salinity result in more dissolved oxygen [44].

**Fig. 4:**
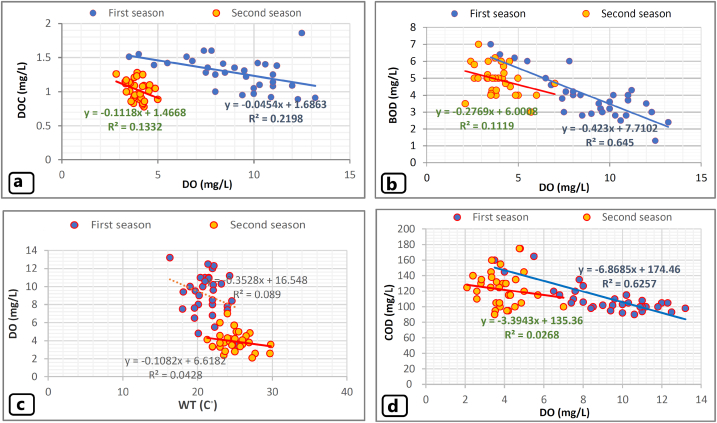
a: represents a relationship between DO and DOC, b: represents the relationship between DO and BOD, c represents a relationship between DO and WT (C), d represents a relationship between DO and COD.

The reverse relationships between the DO and DOC, BOD, water temperature (W.T. C^◦^), COD were clear in the results of groundwater for two sampling seasons (Fig. 4 a, b, c and d). The increase in BOD in LZR for the second season is quite reflected in the increase in organic pollutants in the river in this season which was also clear with DO decrease during this season. The recorded DO values in the second season were less than DO values that were recorded in the first season, whereas BOD values for the second season, in general, were higher than The BOD values for the first season (Table 2).

**Fig. 5:**
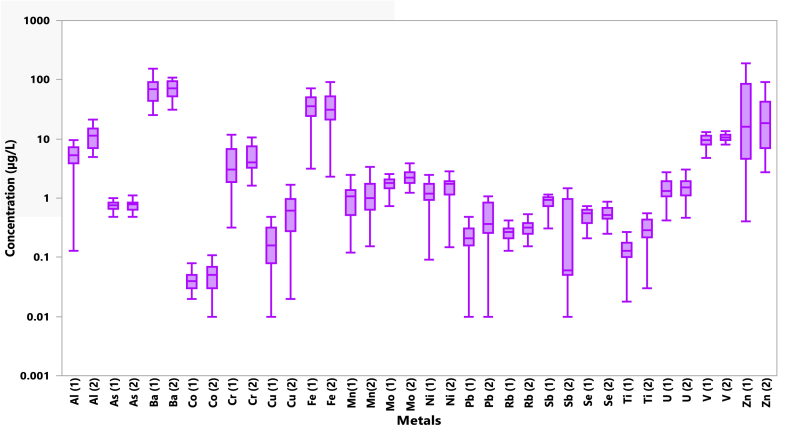
Boxplots for the heavy metals concentrations during the first season expressed as metal (1) and second season expressed as metal (2), The horizontal purple lines (inside the boxes) denote the medians of the concentrations. The bottom and top of the box show the first and third quartiles (Q1 and Q3). (For interpretation of the references to color in this figure legend, the reader is referred to the Web version of this article.)

#### 3.1.2 Heavy metals

A summary of statistical results of ranges, means, and standard deviations of groundwater samples and LZR samples during the first and second seasons are presented in Table 3. On the other hand, the detailed results of heavy metals are displayed in Figure (5). The average abundance of the heavy metals in groundwater follows the decreasing order of: Ba > Zn > Fe > V > Al > Cr > Mo > U > Ni > Mn > Sb > As > Se > Cu > Pb > Rb > Ti > Co > Sn > Cd > Th for the first season and follows the decreasing order of: Ba > Zn > Fe > Al > V > Cr > Mo > Ni > U > Mn > As > Cu > Se > Pb > Sb > Ti > Rb > Co > Sn > Cd > Th for the second season (Table 3).

The most affected groundwater samples by the land uses are W2, W7 and W31. The highest solute concentrations were recorded in groundwater sample W2. This well was directly affected by waste spills from a petroleum **refinery** that was located too close to well W2. whereas the causes behind the increased solute concentrations in wells W7 and W31 are the effect of effluents to groundwater from domestic sewage from unsealed septic tanks for the houses in the rural areas which are too close to these wells. The results of the hydrochemical analysis showed a slightly increasing in solutes in wells W19, W18, and W12 (during the second season), this resulted, from excessive agricultural practices such as the application of fertilizers. Besides, well W12 could be affected by seepages from unsealed artificial ponds of fish farming near the location of well W12.

### 3.2 Hydrogeochemical processes

#### 3.2.1 Saturation state of groundwater result

**Fig. 6:**
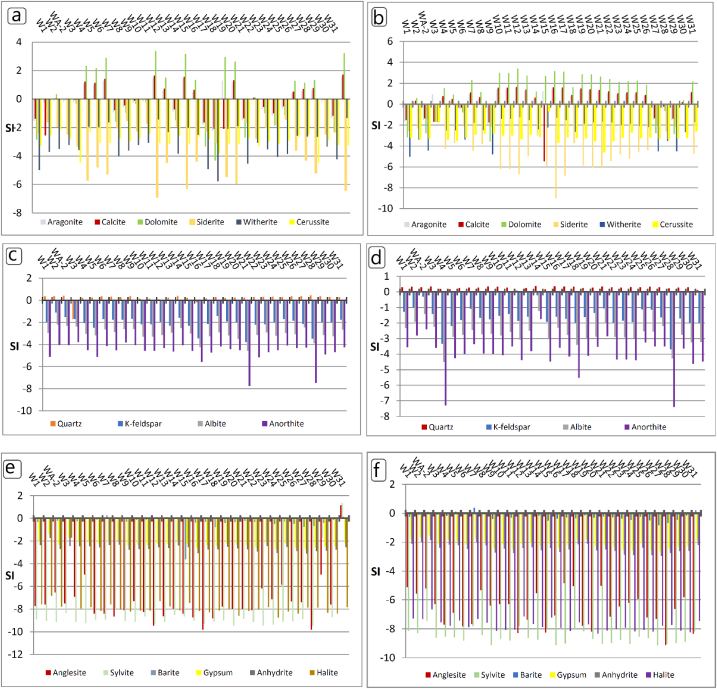
Distribution of SI for each of carbonate minerals (a& b) during the first and second seasons respectively, silicate minerals (c& d) during the first and second seasons respectively, and halide and sulfate minerals (e& f) during the first and second seasons respectively.

Alkalinity, Ph, WT, ORP, DO, cations, anions and heavy metals all were used in the input of PHREEQC software to get the saturation states of groundwater. The descriptive statistic results of SI of groundwater for two sampling seasons are presented in Table 4, while the detailed values of SI are shown in Fig. 6. Moreover, Log PCO_2_ is also calculated. The partial pressure of CO_2_ (pCO_2_) is determined from geochemical speciation modeling. The log PCO_2_ is equal to the value printed for the saturation index of CO_2_ (g) in the PHREEQC output (Table 4). The results suggest different mineral–solution interactions and precipitation–dissolution. Groundwater samples taken during the first season were supersaturated in carbonate minerals of aragonite for most samples with a percent of 84.3%, except for samples W1, WA-2, W27, W28 and W29, and calcite and dolomite for most samples except (W1, WA-2, W3, W6, W8, W27, W28 and W29) (Fig. 6 a &b).

**Table 4:** Descriptive statistic of SI in groundwater for the first and the second seasons.

SI	Chemical Composition	First season				Second season			
		Min	Max	Mean	SD	Min	Max	Mean	SD
Carbonate Minerals									
**Calcite**	CaCO_3_	−5.47	1.61	0.27	1.72	−2.55	1.72	−0.09	1.17
**Aragonite**	CaCO_3_	−1.89	1.51	0.47	1.04	−2.23	1.57	−0.05	1.04
**Cerussite**	PbCO_3_	−4.58	−0.68	−3.08	0.62	−4.49	−2.16	−3.01	0.51
**Dolomite**	CaMg (CO_3_)_2_	−3.59	3.4	1.05	0.90	−4.32	3.38	0.14	2.10
**Siderite**	FeCO_3_	−9.03	−1.43	−4.30	0.67	−6.89	−1.41	−3.41	1.72
**Witherite**	BaCO_3_	−5.03	−1.25	−2.50	0.45	−5.76	−1.34	−3.15	1.09
Quartz and Feldspar minerals									
**Quartz**	SiO_2_	−1.7	0.44	0.21	4.04	0.1	0.38	0.28	0.06
**Albite**	NaAlSi_3_O_8_	−4.57	−2.2	−3.04	0.51	−4.51	−1.23	−2.72	0.65
**Anorthite**	CaAl_2_Si_2_O_8_	−7.75	−3.79	−4.67	0.86	−7.39	−1.74	−4.01	1.12
**K-feldspar**	KAlSi_3_O_8_	−3.79	−1.1	−2.07	2.67	−3.69	−0.21	−1.65	0.67
Halide and Sulfate minerals									
**Halite**	NaCl	−8.74	−1.7	−7.79	7.65	−8.33	−6.66	−7.76	0.39
**Sylvite**	KCl	−9.48	−1.7	−8.7	6.85	−9.14	−7.46	−8.59	0.43
**Anglesite**	PbSO_4_	−9.81	−4.97	−7.59	1.15	−9.13	−4.84	−6.69	1.16
**Barite**	BaSO_4_	−3.6	0.28	−0.32	0.64	−0.82	0.39	−0.21	0.25
**Anhydrite**	CaSO_4_	−3.1	−1.76	−2.63	0.26	−2.95	−1.86	−2.45	0.28
**Gypsum**	CaSO4·2H2O	−2.75	−1.4	−2.27	4.67	−2.64	−1.55	−2.14	0.28
**CO**_**2**_**(g)**	CO_2_(g)	−6.09	−1.55	−3.49	2.15	−4.55	−1.29	−2.88	0.98

Groundwater samples were undersaturated with other carbonate minerals such as cerussite, siderite, and witherite (Fig. 6 a &b). While the groundwater samples for the second season were supersaturated with carbonate minerals of aragonite with a percent of 40.6% from samples, calcite with a percent of 37.5%, and dolomite for samples of 46.8% (Fig. 6 a &b). Whereas the rest of the samples were undersaturated. All groundwater samples with respect to cerussite, siderite and witherite minerals were undersaturated (Fig. 6 a &b). The highest values during the second season were detected in W12 and W31. The groundwater samples are undersaturated with PCO_2_ for two sampling seasons (Table 4). The SI of the minerals shows the groundwater samples for the first season can well produce the precipitation of calcite, aragonite, and dolomite minerals in most groundwater samples.

While the remaining groundwater samples show undersaturation of carbonate minerals, this is due to the presence of carbonate as a matrix material in the clastic aquifer, which is considered the main source that reacts to dominate groundwater chemistry, supplying Ca^2+^ and HCO_3_^⁻^ ions.

Quartz mineral was supersaturated in all groundwater samples for both sampling seasons (Fig. 6 c& d) except W3, and W16 in the first season. The feldspar minerals of K-felspar, albite and anorthite were all undersaturated in the dissolution state (Table 4, Fig. 6 c& d). Sodium is mainly derived from weathering of Na-feldspar or any member of the plagioclase solid solution series between albite and anorthite (Ca-feldspar) [45]. Additionally, plagioclase weathering releases Ca^2+^ cation. The stability of the silicate minerals in a groundwater system can be evaluated by calculating the saturation state of the groundwater for a given mineral. For example, for albite, the dissociation reaction is: NaAlSi_3_O_8_ + 4H^+^ + 4H_2_O → Na^+^ + Al_3_^+^ + 3H_4_SiO_4_ [45].

Groundwater samples were undersaturated with respect to halide and sulfate minerals (Table 4, Fig. 6 e& f), Except barite at W7 and W31, Besides, anglesite and sylvite in W31 for the first season. In the second season, the barite mineral is supersaturated in W5, W7 and W31(Fig. 6 e& f). The groundwaters have distinctly higher calcium and sulfate concentrations which have been attributed to the dissolution of gypsum (CaSO_4_·2H_2_O) [46]. The abundance of silicate minerals that are primarily included within the ambient rock materials of the tertiary and quaternary clastic aquifer system in the study region. The undersaturation in most minerals of feldspars, and halides, sulfates could provide the solution with significant amounts of Ca^2+^, Na^+^, Mg^2+^, HCO_3_^⁻^, Cl^⁻^ and SO_4_^2⁻^ ions.

#### 3.2.2 Rock–water interaction

**Fig. 7:**
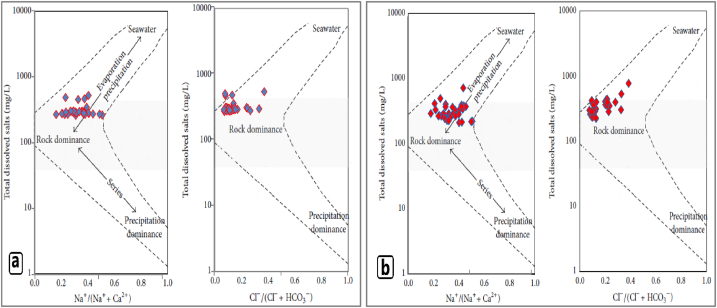
Gibbs plot of groundwater samples (a) for the first season, and (b) for the second season.

Reactions between groundwater and aquifer minerals have a significant role in groundwater [46]. In regions of dry and semiarid climatic conditions, evaporation may also contribute to water chemistry. Plotting the major ionic constituents of groundwater samples of the area in Gibbs plot [38] showed that the samples fall in the field of rock weathering dominance, indicating that the weathering of rocks primarily controls the major ion chemistry of groundwater in this region (Fig. 7 a & b). The evaporation effect on groundwater of the study area was too limited (Fig. 7 a & b) because the depth of groundwater varied from 15 to 150 m, which led to the groundwater being far from the effect of evaporation. The finding of the Gibbs plot agreed with the finding of the hydrogeochemical model which indicated the source of major ions in most water samples was the rock material of the groundwater aquifer.

#### 3.2.3 Classification of water type

Piper diagram results reveal that the plotted points of the majority of the groundwater samples are classified as earth-alkaline water with an increased portion of alkali with prevailing bicarbonate for the first and second seasons, indicating the high concentrations of Ca^2+^, HCO_3_^−^ which were derived from carbonate minerals that were abundant in rock material of aquifer [17,47]. While the groundwater sample W2 for the two sampling seasons was classified as earth-alkaline water with an increased portion of alkali with prevailing SO_4_^2⁻^ and Cl^⁻^.

**Fig. 8:**
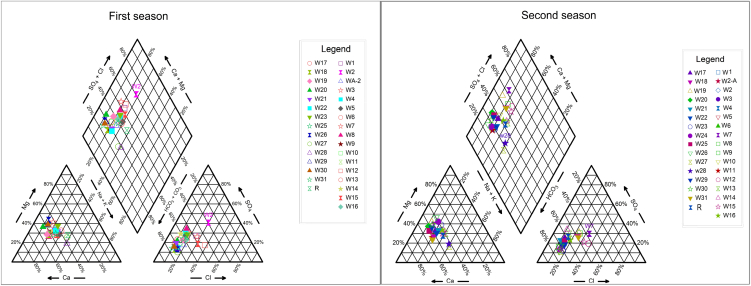
Piper diagram of water samples for the first and second seasons.

The increase in SO_4_^2⁻^ and Cl^−^ ions in the W2 sample indicated the effect of the petroleum refinery located close to the well site, the infiltration of the untreated discharges from the refinery led to the pollution of groundwater which led to an increase in SO_4_^2⁻^ and Cl^−^ concentrations also the W27 and W28 samples for two sampling seasons were classified as alkaline water with prevailing of bicarbonate (Fig. 8).

### 3.3 Suitability for human drinking

The fundamental physical, chemical, biological characteristics, major ions, minor ions and heavy metals are compared with IQS (2009) and WHO (2008, 2017) to evaluate the suitability of the Shwan sub-Basin for human drinking. The results show that most of the measured parameters (Table 2, Table 3) were not exceeded the standard limits and most groundwater and LZR samples were suitable for human drinking according to the quality guidelines (IQS 2009, WHO 2008 and 2017). There were some exceptions with respect to BOD that exceeded both quality guidelines in groundwater samples W2, W7, W12, W18 and W19 for the first season and groundwater samples W12, W13, W14, W16, W17, W21, W22, W24, W25, W27, W28, W29, W30, W31 and LZR sample (R) for the second season (Table 2). Besides, turbidity exceeded quality guidelines for both seasons except for a few numbers of samples that were within these guidelines. COD also exceeded the quality guidelines in all samples. PO_4_ slightly exceed quality guidelines in W2, W7 and W18 for the first season, and slightly exceeded quality guidelines in W2, W7, W15, W18 and W31 for the second season. Most of the heavy metals were within global quality guidelines except, Al exceeded the IQS (2009) and WHO (2008, 2017) guidelines in LZR during the first season and W14 during the second season. V exceeded the WHO (2017) guideline in most groundwater samples except W21, W25 and LZR for the first season, and W25 and LZR for the second season. Fe exceeded the guideline in W13 and LZR for the first season. Mn exceeded both guidelines in the LZR sample for the first season. Zn exceeded the IQS (2009) guideline in W13 and W29 for the first season and W14 for the second season samples.

Fe exceeded the guideline in W13 and LZR for the first season. Mn exceeded both guidelines in LZR sample for the first season. Zn exceeded the IQS guideline in W13 and W29 for the first season and W14 for the second season samples.

**Fig. 9:**
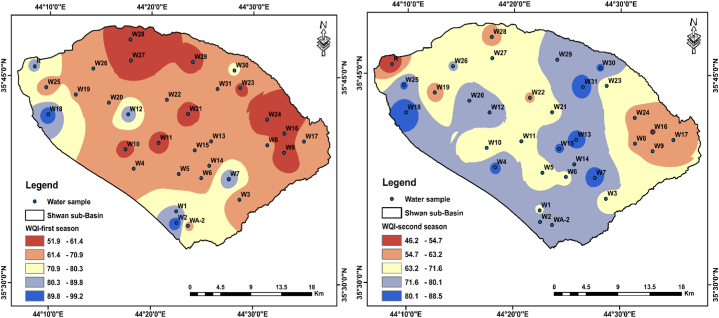
Spatio-temporal maps of WQI values in water samples for two sampling seasons.

The results of the WQI show that the most of water samples were poor to very poor in quality for both seasons (Table 5), except the water sample from LZR for the second season showed good water quality (Table 5). The spatial-temporal distribution of WQI values in all water is displayed in Fig. 9 which shows the water samples from the western and southern sides of the sub-Basin have the worse WQI.

**Table 5:** WQI values and water types of the samples.

First season				Second season			
Sample	WQI	Sample	WQI	Sample	WQI	Sample	WQI
**W1**	**80.6**	**W17**	**65.9**	**W1**	**70.2**	**W17**	**61.7**
**W2**	**99.2**	**W18**	**94.8**	**W2**	**79.7**	**W18**	**88.5**
**WA-2**	**67.6**	**W19**	**70.6**	**WA-2**	**79.6**	**W19**	**58.3**
**W3**	**63.5**	**W20**	**63.6**	**W3**	**68.6**	**W20**	**77.5**
**W4**	**67.2**	**W21**	**58.2**	**W4**	**82.0**	**W21**	**69.1**
**W5**	**62.7**	**W22**	**64.3**	**W5**	**63.9**	**W22**	**62.0**
**W6**	**67.6**	**W23**	**59.8**	**W6**	**69.1**	**W23**	**65.1**
**W7**	**90.7**	**W24**	**56.1**	**W7**	**85.7**	**W24**	**60.9**
**W8**	**64.4**	**W25**	**65.9**	**W8**	**62.5**	**W25**	**81.3**
**W9**	**57.9**	**W26**	**62.2**	**W9**	**62.7**	**W26**	**72.1**
**W10**	**57.9**	**W27**	**51.9**	**W10**	**63.5**	**W27**	**63.9**
**W11**	**58.4**	**W28**	**54.1**	**W11**	**68.2**	**W28**	**61.5**
**W12**	**87.2**	**W29**	**59.6**	**W12**	**76.2**	**W29**	**74.0**
**W13**	**64.6**	**W30**	**73.0**	**W13**	**83.1**	**W30**	**80.9**
**W14**	**64.6**	**W31**	**69.5**	**W14**	**78.1**	**W31**	**86.1**
**W15**	**64.5**	**R**	**81.7**	**W15**	**81.2**	**R**	**46.2**
**W16**	**53.4**			**W16**	**53.9**		
**Min**	51.9			**Min**	53.9		
**Max**	99.2			**Max**	88.5		
**Mean**	66.8			**Mean**	71.6		
* Good * Poor *Very poor

The variation in WQI between the two sampling seasons was due to the effect of the increased solute concentration during the second season which led to a shift of the samples WA-2, W4, W13, W14, W15, W20, W25, W30 and W31 from poor to very poor water quality (Table 5, Fig. 9). The interpretation of the increased solute concentrations during the second season was the effect of the climatic condition. e.g., low amount of precipitation which resulted in limited groundwater recharge. Thus, the concentrations of dissolved ions increased in groundwater by interaction with aquifer rock material. In addition, the low groundwater recharge led to increasing pollutant concentrations in groundwater [48]. However, this effect was clear at wells that were affected by petroleum refinery discharge (W2) and the wells close to domestic septic tanks.

## 4 Conclusions

This study indicated that the hydrogeochemical process of rock weathering is the main process controlling the concentrations of solutes in groundwater. From the hydrochemical and geochemical processes. However, we conclude that the soluble ions in waters mainly derive from aquifers matrix and soil weathering, partly from the precipitation input and land use activity. The majority of the hydrochemical ions were derived from the minerals that comprise the aquifer's rock, and these ions have been used to characterize the hydrochemistry and water quality of groundwater. The consequences of the dry weather conditions by high air temperatures, high evaporation, as well as very limited precipitation during the study period led to low groundwater recharge, Hence, in such condition's anticipation of elevated solute concentrations from dissolution processes of the groundwater during the resident time and along its flow path, consequently the raising in solutes concentrations in groundwater in the second season than those in the first season. Thus, the groundwater quality during the second season was worse than the groundwater during the first season. The study revealed that wells from the eastern parts of the sub-Basin are not affected by land use and land cover which is mainly agricultural activities, grazing of animals and a few barren regions. This could be interpreted from the limited use of fertilizers in this part besides the and the higher depth of groundwater compared with the depth of the groundwater in the rest wells of the sub-Basin. Whereas the southern and western part of the sub-Basin shows in general higher solute concentrations. Industrial activities from a petroleum refinery, as well as building block and paint factories, are considered the most influential activities in the southern part of the sub-Basin. The agricultural activities, as well as the effluents from domestic sewage, were considered the land use practices behind the high levels of solutes from the wells in this western part of the sub-Basin. This study characterized the hydrogeochemical processes in late Pliocene and Quaternary clastic aquifers of groundwater that will be a good reference for future studies in northern Iraq that extract the groundwater from aquifers resembling geological and lithological settings. In addition, the finding of the land use effect on groundwater quality would help to identify the pollution levels and sources in the study area that we recommend reducing the use of fertilizers and establishing treatment units in a petroleum refinery to prevent any untreated discharges from this refinery, as well as the wells that detected with poor water quality requiring immediate interventions for the sustainability of the sub-Basin water resources.

## Author contribution statement

Hind Fadhil AL-Gburi: Conceived and designed the experiments; Performed the experiments; Analyzed and interpreted the data; Contributed reagents, materials, analysis tools or data; Wrote the paper.Balsam Salim Al-Tawash; Omer Sabah Al-Tamimi: Conceived and designed the experiments; Performed the experiments; Analyzed and interpreted the data; Wrote the paper.Christoph Schuth: Performed the experiments; Analyzed and interpreted the data; Contributed reagents, materials, analysis tools or data; Wrote the paper.

## Funding statement

This research did not receive any specific grant from funding agencies in the public, commercial, or not-for-profit sectors.

## Data availability statement

Data included in article/supplementary material/referenced in article.

## Declaration of competing interest

The authors declare that they have no known competing financial interests or personal relationships that could have appeared to influence the work reported in this paper.
